# A case report of uterine necrosis following cesarean section: lessons learned from cross binding suture for refractory postpartum hemorrhage

**DOI:** 10.3389/fmed.2025.1568361

**Published:** 2025-03-12

**Authors:** Sheng-ying Yang, Yang Liu, Zi-jun Zhou, Jon-bo-wen Yan, Tao Li, Bao-yu Cao, Jun-qiang Li

**Affiliations:** Department of Obstetrics and Gynecology, Affiliated Hospital of Southwest Jiaotong University, The Third People’s Hospital of Chengdu, Chengdu, China

**Keywords:** postpartum hemorrhage, uterine necrosis, cesarean section, B-Lynch suture, uterine compression suture, hysterectomy

## Abstract

Uterine necrosis following cesarean section is an extremely rare but serious complication. This case report presents a unique scenario where a cross binding suture technique was employed to manage intractable postpartum hemorrhage (PPH), ultimately leading to uterine necrosis and hysterectomy. The case underscores the challenges of managing severe PPH and highlights the potential complications of unconventional surgical techniques. It also emphasizes the importance of early recognition and intervention to minimize maternal morbidity and mortality.

## Introduction

According to the statistics of the WHO, postpartum hemorrhage (PPH) is the leading cause of maternal mortality, accounting for about 27% of the total number of maternal deaths (1). PPH is a common obstetric complication. According to the “Guidelines for the Prevention and Management of PPH” (2023 edition), PPH is defined as a blood loss of ≥500 mL within 24 h after vaginal delivery or ≥ 1,000 mL after cesarean section (2). Many countries consider blood loss exceeding 1,000 mL as intractable PPH. Pharmacotherapy is the preferred method for PPH, but in many cases, other interventions are also required, especially when bleeding is severe, necessitates blood transfusion, or leads to hemodynamic instability. Conservative techniques are initially used to control PPH and avoid hysterectomy, including uterine balloon tamponade, gauze packing, uterine compression sutures, uterine artery ligation, and uterine artery embolization. So far, there is no evidence that one method is superior to another (3). This article discusses one of the compression suture techniques, namely the case of uterine necrosis and hysterectomy caused by refractory PPH treated with uterine cross-binding suture combined with ligation of the uterine artery ascending branches, and summarizes and reflects on different hemostatic methods to provide value for clinical decision-making.

## Case presentation

**Figure 1:**
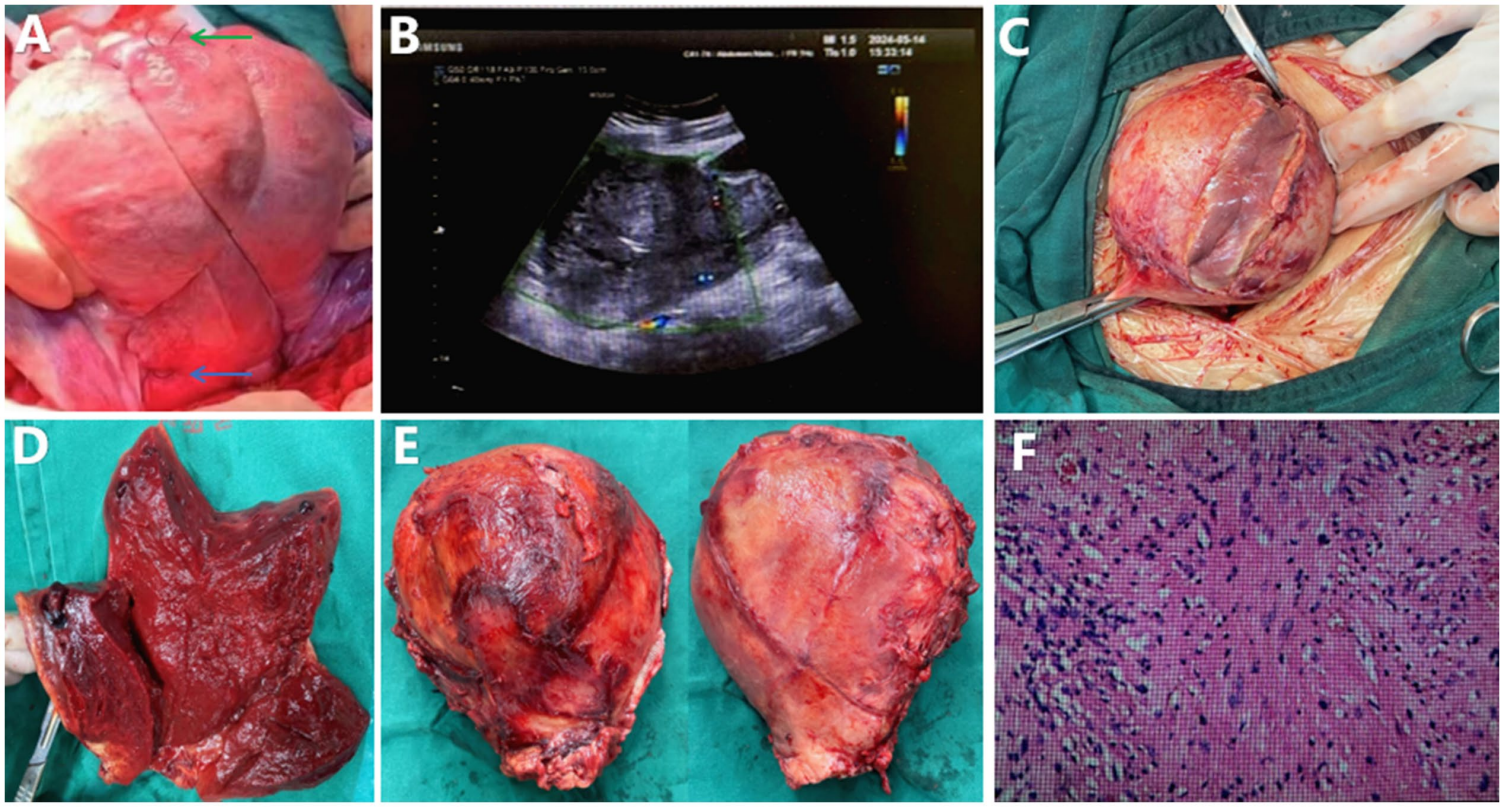
**(A)** Shows the intraoperative image after cross-binding suture during cesarean section, with the green arrow indicating the knot at the fundus and the blue arrow indicating the ligation site of the uterine artery ascending branch. **(B)** Presents the color doppler ultrasound image of the uterus taken 2 weeks postoperatively, showing minimal blood flow signals, suggesting uterine necrosis. **(C)** Shows the edematous and friable uterine myometrium observed during laparotomy. **(D)** Displays the cross-sectional view of the removed uterus, revealing ischemic necrosis of the tissue. **(E)** Illustrates the removed uterus, with the absorbable suture from the cross-binding suture integrated into the uterine serosal layer. **(F)** Shows the hematoxylin and eosin stained section of the removed uterus at 40x magnification, revealing extensive necrosis with surrounding acute inflammation.

The patient was a 27-year-old primiparous woman at 39 weeks of gestation, and there was no obvious abnormality in antenatal examination. She was transferred to a cesarean section due to arrest of the second stage of labor during a vaginal delivery attempt. During the surgery, intractable PPH occurred, prompting the use of a uterine cross-binding suture and ligation of the bilateral uterine artery ascending branches (Figure 1A). Prior to the cross-binding suture, standard pharmacological and mechanical interventions were administered. However, uterine bleeding persisted, and bleeding exceeded 1,000 mL (combination of volumetric and gravimetric methods), necessitating surgical intervention. Hemoglobin levels decreased from 114 g/L preoperatively to 82 g/L intraoperatively, as confirmed by repeated laboratory tests. On the first postoperative day, the patient developed abdominal pain and fever, with a peak temperature of 39.5°C. Physical examination revealed tenderness and rebound tenderness in the lower abdomen. Laboratory tests, including complete blood count, C-reactive protein, and procalcitonin, indicated elevated infection markers. Empirical treatment with meropenem was initiated for anti-infective therapy.

After 3 days of treatment with no improvement and the development of abdominal muscle rigidity, a gynecological ultrasound was performed. The findings showed an enlarged uterus with an abnormal shape (9.7×9.7×9.3 cm), heterogeneous myometrial echotexture, and a hypoechoic area (6.0×2.5 cm) at the fundus, suggesting accumulated blood with infection. A fluid dark area (0.9 cm in diameter) was observed within the uterine cavity, and a larger fluid dark area (5.4×5.2 cm) was detected in the rectouterine pouch. Abdominal computed tomography (CT) revealed an enlarged uterus with an irregular fundal shape, discontinuous myometrial layer, mixed density shadows, and a small amount of gas density shadows within the uterine cavity and at the fundus, suggesting infectious lesions and intestinal obstruction, as well as thickening of the right retroperitoneum.

Subsequent cultures of endometrial and vaginal secretions identified multiple bacteria, including *Mycoplasma hominis*, *Ureaplasma urealyticum*, *Enterococcus faecalis*, and *Escherichia coli*. Despite 2 weeks of anti-infective treatment based on antibiotic sensitivity tests, the infection was not controlled. Follow-up ultrasound showed almost no blood flow signals in the uterus, suggesting necrosis (Figure 1B). A repeat Abdominal CT scan demonstrated slight uterine size reduction, irregular fundal shape, discontinuous myometrial layer, mixed density shadows, and gas density shadows, indicating liquefactive necrosis and infectious lesions. There were also signs of peritonitis, with increased fluid in the pelvic and abdominal cavities compared to previous findings.

Considering the ineffective anti-infective treatment, persistent high fever, and the possibility of uterine infection and necrosis, an exploratory laparotomy was performed. Intraoperatively, ischemic necrosis of the uterus was confirmed. Severe adhesions in the pelvic and abdominal cavities were observed, with the uterus enlarged to the size of a 4-months pregnancy, showing significant edema. Multiple abscesses were present in the anterior myometrial layer, emitting a foul odor, and the tissue was edematous and friable. During the emergency laparotomy, written consent was obtained from the patient’s spouse after explaining the necessity of hysterectomy, along with verbal consent from the patient herself, who remained conscious under spinal anesthesia. Written consent was subsequently obtained from the patient postoperatively. This process adhered to institutional protocols for surgical procedures. The removed uterus measured approximately 17 × 8 × 8 cm, with suture lines from the cross-binding suture on both the anterior and posterior walls integrated into the uterine serosal layer (Figures 1C–E). Postoperative pathological examination revealed multiple extensive necrosis within the myometrium of the uterine body, accompanied by surrounding acute inflammation, and the presence of inflammatory granulation tissue within the uterine cavity (Figure 1F).

After the surgery, the patient underwent an extended 2-week course of anti-infective treatment before recovering and being discharged from the hospital.

## Discussion

Currently, widely recognized methods for controlling postpartum hemorrhage include uterine cavity packing with gauze, uterine balloon tamponade, and uterine compression sutures, among others. Uterine compression sutures are a simple, safe, and effective method for controlling postpartum hemorrhage in emergency situations while preserving fertility (4). The B-Lynch suture is a widely recognized method of uterine compression suture. The suture begins below the right inferior limit of the uterine incision, inserting the needle through the ante-rior uterine wall. The needle is exteriorized again in the an-terior uterine wall above the superior border of the incision. The thread then goes to the uterine posterior wall, passing above the fundus, and then the needle is inserted through the posterior wall in a point corresponding to the thread’s anterior exit. Afterwards, the needle should be inserted a few centimetres laterally to the entry point, and the thread is then passed longitudinally through the fundus to the ante-rior wall. The needle is then inserted above the left superior margin of the uterine incision through the uterus wall and exits on the inferior margin (parallel to the first stitches). An assistant should compress the uterus while the surgeon pulls the thread firmly and applies a strong kno (3). With the advancement of surgical techniques, numerous variations of uterine compression sutures have evolved from the B-Lynch suture (3, 5–7). The first step in treating intractable postpartum hemorrhage with the cross-binding suture technique is to apply manual compression to the uterus with both hands, observing whether the amount of uterine bleeding decreases to assess the likelihood of successful hemostasis with the uterine binding suture. Monocryl absorbable suture and round needles are used. The entry point is parallel to the peritoneal reflection and away from the area where the uterine artery is located. The suture passes through the avascular area of the left broad ligament and is brought around to the opposite fundus. Manual compression is applied downward and inward on the fundus and body of the uterus with both hands, while the surgeon tightens both ends of the suture and ties a knot 2 cm away from the right cornua at the fundus. The same procedure is performed on the right side. After both cross-binding sutures are tied, the two suture knots are then tied together at the fundus (Figure 1A) (7). However, compression sutures carry risks such as endometritis, pyometra, intrauterine adhesions, and uterine necrosis (3, 4, 8, 9). Uterine compression sutures are often included as a second-line intervention for managing PPH in guideline (10). Compression sutures are an important fertility-sparing measure in cases of PPH, but regardless of the type of suture used, there is a risk of postoperative uterine necrosis (11). We present a case of uterine necrosis leading to hysterectomy following a modified uterine cross-binding suture. Therefore, we recommend that compression sutures be used with caution. Given the risk of complete uterine necrosis, other hemostatic methods, such as uterine cavity packing with gauze, should be considered first. Some studies have found that uterine cavity packing with gauze has a better hemostatic effect than uterine balloon compression, and it is associated with a lower incidence of complications. Chitosan covered gauze is a promising treatment option for PPH, which can significantly reduce the incidence of postpartum bleeding with almost no severe complications, and is an effective means of treating postpartum hemorrhage (4).

In this case, we believe that uterine necrosis is caused by the dual effects of infection and ischemia. Although the patient did not have premature rupture of membranes and was given cefazolin before and after surgery to prevent infection, prolonged second stage of labor, repeated vaginal examinations, and intrapartum cesarean section were all high-risk factors for retrograde infection. Cultures identified *Mycoplasma hominis*, *Ureaplasma urealyticum*, *Enterococcus faecalis*, and *Escherichia coli*—organisms commonly associated with ascending genital tract infections (12). Ischemia and infection likely acted synergistically to accelerate necrosis. Compromised blood flow from the compression sutures and arterial ligation impaired tissue oxygenation and immune response, while bacterial proliferation exacerbated inflammation and microvascular thrombosis (13, 14). This case underscores the imperative for clinicians to rigorously mitigate risk factors when managing postpartum hemorrhage. In scenarios involving multiple high-risk elements—such as prolonged labor, intrapartum cesarean section, and devascularization procedures—the implementation of uterine compression sutures warrants cautious deliberation.

## Conclusion

The cross binding suture technique, while effective in controlling bleeding in some cases, can lead to severe complications such as uterine necrosis. The ischemia caused by the tight compression of the uterine vessels can result in tissue death. Early recognition of ischemic symptoms and prompt intervention are crucial. In this case, the delay in recognizing the signs of uterine ischemia contributed to the progression to necrosis. This case serves as a reminder of the potential risks associated with unconventional surgical techniques in managing intractable PPH. It emphasizes the need for a multidisciplinary approach, including obstetricians, anesthesiologists, and surgeons, to manage such cases. Further research is needed to develop safer and more effective methods for controlling severe postpartum bleeding.
